# What matters most to patients following percutaneous coronary interventions? A new patient-reported outcome measure developed using Rasch analysis

**DOI:** 10.1371/journal.pone.0222185

**Published:** 2019-09-05

**Authors:** Sze-Ee Soh, Anna L. Barker, Darshini R. Ayton, Susannah Ahern, Renata Morello, Jeffrey Lefkovits, Angela L. Brennan, Susan Evans, John R. Zalcberg, Christopher M. Reid, John J. McNeil

**Affiliations:** 1 Department of Epidemiology and Preventive Medicine, Monash University, Melbourne, Victoria, Australia; 2 Department of Physiotherapy, Monash University, Melbourne, Victoria, Australia; 3 NHMRC Centre for Research Excellence in Cardiovascular Outcomes Improvement, Curtin University, Perth, Western Australia, Australia; University of Birmingham, UNITED KINGDOM

## Abstract

**Introduction:**

Measuring patient reported outcomes can improve the quality and effectiveness of healthcare interventions. The aim of this study was to identify the final set of items that can be included in a patient-reported outcome measure to assess recovery of patients following percutaneous coronary interventions.

**Methods:**

A consecutive sample of 200 patients registered in the Victorian Cardiac Outcomes Registry participated in a telephone survey 30 days following their percutaneous cardiac procedure. Rasch analysis was used to select the best set of items to form a concise and psychometrically sound patient-reported outcome measure. Key measurement properties assessed included overall fit to the Rasch measurement model, unidimensionality, response formats (thresholds), targeting, internal consistency and measurement invariance.

**Results:**

Five items were identified as being reliable and valid measures of patient-reported outcomes: pain or discomfort, shortness of breath, confidence in performing usual activities, feeling unhappy and having trouble sleeping. Data showed overall fit to a Rasch model of expected item functioning (χ^2^ 16.99; *p* = 0.07) and all items demonstrated unidimensionality (*t*-test less than 0.05 threshold value). Internal consistency was acceptable (equivalent Cronbach’s α 0.65) given there are only five items, but there was a ceiling effect (mean logit score -1.24) with compromised score precision for patients with better recovery.

**Conclusions:**

We identified a succinct set of items that can be used in a patient-reported outcome measure following percutaneous coronary interventions. This patient-report outcome measure has good structural validity and acceptable internal consistency. While further psychometric evaluations are recommended, the items identified capture the patient’s perspective of their recovery following a percutaneous coronary intervention.

## Introduction

Projections of mortality and burden of disease to 2030 indicate that coronary artery disease (CAD) continues to be one of the leading causes of death worldwide [1, 2]. Percutaneous coronary intervention (PCI) is a frequently used medical procedure to manage patients with CAD, including those who have acute coronary syndrome or stable ischemic heart disease [3, 4]. It involves the insertion of a balloon or stent catheter to promote flood flow into the coronary arteries [4]. While there are high levels of survival post procedure, there is limited information about how patients perceive their recovery and quality of life following PCI [5].

Understanding outcomes following health interventions from the patient’s perspective has the potential to improve the quality and effectiveness of healthcare provided by an organisation [6], and can guide clinical decision-making [7, 8, 9]. In the area of ischaemic cardiac disease, three patient-reported outcome measures (PROMs; Seattle Angina Questionnaire, Rose Dyspnoea Score and Patient Health Questionnaire)—were identified by the International Consortium for Health Outcomes Measurement (ICHOM) to quantify cardiac-related symptoms, functional status and quality of life from the patient’s perspective [10–12]. However, these instruments were developed for use in patients with angina or ischemic heart disease and have not been validated for patients following PCI. To date, only the Coronary Revascularisation Outcome Questionnaire–Percutaneous Transluminal Coronary Angioplasty (CROQ-PTCA) [13] can be used in this population, but with 47 items, it has high respondent burden. This highlights a need to develop a brief and concise PROM to assess recovery following PCI procedures that is quick and easy to administer in the clinical setting including clinical quality registries.

PROMs may be incorporated into an existing data collection within clinical registries where they provide complementary information to routinely collected clinical outcomes data to support quality of care [6, 14]. International examples of registries that have integrated PROMS include prostate cancer registries, the British Spine Registry, and the National Health Service (NHS) in the United Kingdom [15]. The Victorian Cardiac Outcomes Registry (VCOR) is a state-wide population-based clinical quality registry that provides benchmarking data on the outcomes of cardiac interventions such as PCI in Victoria, Australia [3]. VCOR monitors the performance of health services in Victoria, Australia by collecting data about patients undergoing PCI and follows-up on medical outcomes and complications 30-days after the patient has been discharged from hospital.

The 5-item Euroqol (EQ-5D) is currently being used by VCOR to assess perceived health-related quality of life (HRQOL) 30 days post-PCI [3]. While the EQ-5D has been shown to be valid in patients who have experienced unstable angina or myocardial infarction [16, 17], it has not been validated for use in patients following a PCI. In addition, the dimensions of the EQ-5D were selected by members of the EuroQoL group [18]. Previous studies have shown that patients and health care professionals rank the importance of health outcomes differently [19]. In order to truly capture the patient’s perspective of recovery following PCI, the patient should be involved in the identification of domains, outcomes and item wording [20]. Thus, a PROM specific to this population developed by genuine patient-centred methods that can be administered within VCOR is warranted.

Rasch analysis, which is based on latent-trait modelling, is increasingly recognised as the preferred method to construct new questionnaires and the development of PROMs [21]. It is a unique form of item response theory that tests a measure such as a PROM against a mathematical model that is consistent with the key principles of good measurement [22–24]. The advantage of this approach over classical test theory approaches is that it compares the response patterns of individuals to the entire sample to estimate person ‘ability’ and item ‘difficulty’ [23, 25]. When responses to the items corresponds to the Rasch model expectations, ordinal scores can be converted into interval level measures [23, 26]. This will allow percentage change scores to be calculated as the magnitude of separation between scores is provided [27]. Individual item scores for an interval level measure can also be summed to generate a total score, which can be used in parametric statistical analyses [23]. If the data does not fit the model, the outcome scale can be modified based on results of the analyses such as removing items or adjusting the response options [26]. As such, Rasch analysis was considered to be an appropriate approach to evaluate the structural validity of a PROM.

Recently, we conducted a mixed-methods project to identify outcomes most important to patients following PCI [20, 28, 29]. The primary aim of this study was to identify the final set of items that can be included in a PROM (the Monash University cardiac PROM [MC-PROM]) to assess recovery following PCI using the outcomes generated from previous studies. Specifically, we wanted to examine structural validity and whether:

1. The items measured one underlying construct (unidimensionality) and can be summed to provide an overall score;
2. Participants were able to consistently distinguish between the response options of never, rarely, sometimes, often and always (thresholds);
3. The items were inter-related (internal consistency) and able to separate participants across different levels of recovery post procedure;
4. Different groups within the sample (e.g. males versus females, emergency versus elective procedures), despite equal levels of the underlying characteristics being measured, responded differently to an individual item (measurement invariance); and
5. The items were appropriately targeted for the clinical population (floor and ceiling effects).

We also tested convergent validity by examining the correlation of the final MC-PROM items with the EQ-5D, and obtained information on the feasibility and acceptability of the items to ensure that the PROM can be administered effectively within a clinical registry.

## Methods

### Development of the patient-reported outcome measure

This study was the final stage of a larger mixed-methods project (S1 Fig) that included a literature review, focus groups and interviews, and a discrete choice experiment (DCE) [20, 28, 29]. The methodology and results for these studies have been described in detail elsewhere and are briefly summarised below [20, 28, 29].

#### Stage 1: Literature review

A comprehensive and systematic search of peer-reviewed literature was undertaken to identify existing PROMs following elective coronary revascularisation procedures, and to assess the level of patient involvement in the development of these measures [29]. The review identified 27 multidimensional and unidimensional PROMs that can be used in elective coronary revascularisation, as well as 430 symptoms and feelings that were used in the next stage of the project [29].

#### Stage 2: Focus groups and interviews

Eight focus groups and five interviews were conducted to explore patient perceptions of recovery with 32 patients who had undergone an elective or emergency PCI in the last six months [20]. Participants identified 10 symptoms and feelings to be important outcomes post-procedure (S1 Table) [20], which were subsequently confirmed by an expert panel consisting of cardiologists, nurses, health services researchers and allied health professionals[28].

#### Stage 3: Discrete choice experiment

In order to identify the physical, psychological and functional outcomes patients perceived as important following a PCI, a DCE was conducted with 138 people who had undergone the procedure in the last six months [28]. The DCE consisted of 240 choice sets of two health outcome scenarios of the 10 symptoms and feelings described in S1 Table (refer to Barker *et al* [28] for details). For each choice set, the participant was asked to select the scenario they would prefer to experience 30 days post-PCI (S2 Fig). The DCE analysis identified eight symptoms and feelings that were most valued by patients after having a PCI (S3 Fig)[28].

### Refinement of the Monash University cardiac patient-reported outcome measure

#### Setting

Potential public and private Victorian hospitals were identified by VCOR and formally invited to participate in this study. Three tertiary public hospitals and a large private hospital in metropolitan Victoria, Australia were recruited to ensure that a representative sample was obtained. The hospitals ranged in size from moderate (150–500 beds) to large (>600 beds), and have contributed to VCOR since 2013.

#### Participants

Our sample consisted of consecutive VCOR patients aged over18 years who had undergone a PCI at one of the participating hospitals between March and June 2017. No other exclusion criteria were specified. A VCOR Participant Information Statement is provided to all patients on admission and an opt-out procedure is in place for this registry. After completion of the standard VCOR clinical follow-up questions, patients were invited to complete the eight items identified by the DCE as part of their routine telephone follow-up. Thus, if patients completed the eight items during the telephone survey, it was assumed that they agreed and consented to participate in this study. The project was approved by the Monash University Human Research Ethics Committee (MUHREC CF16/26-2016000012) and all relevant participating hospitals including Epworth HealthCare (EH2016-54), Barwon Health (16/42), Monash Health (HREC 16125L) and Melbourne Health (2016.084).

#### Data collection

The eight items identified by the DCE were administered via a telephone survey as part of routine VCOR follow-up 30 days post procedure by the participating hospital. Additional questions were added to the survey to determine the acceptability and feasibility of the items, including item wording and response options from the perspectives of patients and data managers. Data managers were trained on how to administer the survey by a member of the research team. Standard operating procedures were also developed to standardise the way in which the survey was administered. Information on missing data was collected including the reason(s) for missing patient reported outcome data, which could then be used to inform analyses. Study data were collected and managed using REDCap electronic data capture tools hosted at Monash University [30].

#### Measurements

**Fig 1:**
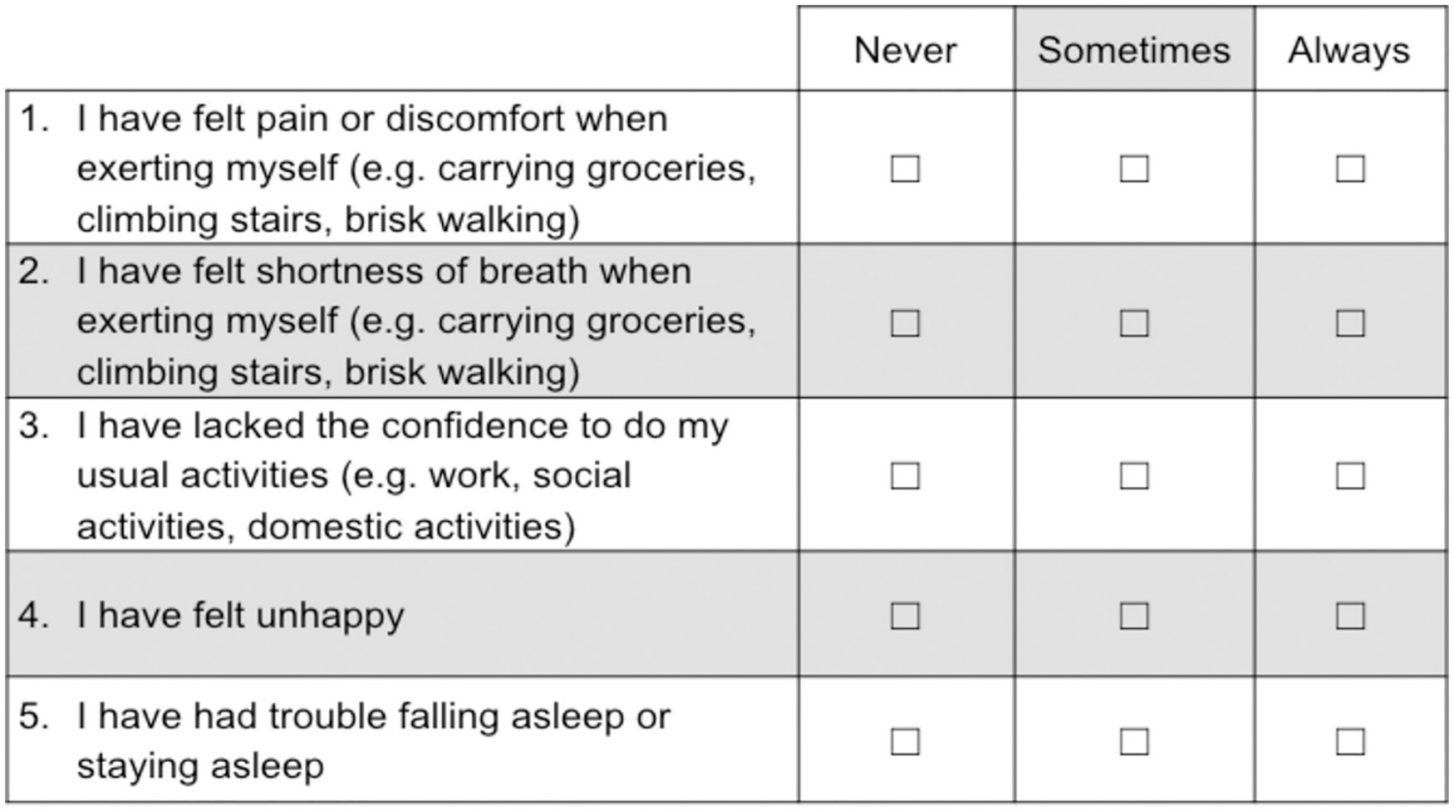
The final Monash University cardiac patient-reported outcome measure (MC-PROM) for patients following percutaneous coronary interventions.

Demographic and clinical information such as age, sex, clinical presentation and HRQOL as measured by the EQ-5D were obtained from data routinely collected by VCOR. The eight patient-reported outcomes that were most valued by patients from the DCE analysis were tested (Fig 1) [28]. Responses to the eight items were recorded using a 5-point rating scale that ranged from never to always, with higher scores representing better recovery. We chose to use a unipolar scale to measure participants’ level of agreement with the outcome because the option of a midpoint or ‘neutral’ with bipolar scales that measure both agreement and disagreement has been shown to contribute to disordered thresholds [22].

### Statistical analysis

Descriptive statistics were used to summarise the characteristics of participants that completed the telephone survey. Reasons for missing data, acceptability responses and feasibility information were also analysed descriptively and reported using frequencies. This included responses from patients regarding the wording of the questions and response options, and the perspectives of data managers to complete the additional items as part of the VCOR 30-day follow-up process. Qualitative participant information such as quotes are presented verbatim.

Item analysis was conducted using Rasch analysis and a pairwise conditional estimating procedure to determine the statistical functioning of the eight items derived from the DCE.

Overall model fit was assessed using three statistics—overall fit, individual person fit and individual item fit—to determine whether the eight items met the expectations of the Rasch measurement model [23]. The χ^**2**^ item-trait interaction statistic was used to assess overall fit, where a non-significant value (*p*>0.05) indicated that the observed data fit the expectations of the Rasch model [22]. Item-person interaction statistics were also examined, where a residual standard deviation (SD) value of ≤1.5 indicated satisfactory fit [22]. Residual fit statistics of individual items and persons were further analysed, where values between ±2.5 indicated adequate fit [22]. Details of the methods and criteria for assessing the measurement properties of the eight items, including statistical tests used, are described in Table 1.

**Table 1:** Methods and criteria to examine key measurement properties of the items generated from previous studies [23–25].

Measurement property	Purpose	Statistical test	Criteria for assessment
Unidimensionality	To assess whether items measure one underlying construct (or concept) so that it can be summed To examine whether the response to one item is dependent on the response to another item (local dependency)	PCA of residuals and equating t-tests Binomial dimensionality test Person-item residual correlation	Two most dissimilar subsets of items identified from PCA, with *p*<0.05 indicating unidimensionality [22]. Where *p*>0.05, unidimensionality supported if lower bounds of CI <0.05 [22]. Local dependency indicated by person-item residual correlation values >0.2 [22, 23].
Response thresholds	To assess if participants had difficulty discriminating between each of the five response options (never, rarely, sometimes, often, always)	Threshold map Category probability curves	Examination of pattern of thresholds When each response option systematically has a point along the location continuum to be the most likely response, thresholds are considered to be ordered.
Internal consistency	To determine the degree of inter-relatedness among items and its ability differentiate participants across different levels of recovery post procedure	Person separation index	Analogous to Cronbach α where PSI values >0.70 indicates good internal consistency reliability [22].
Measurement invariance	To examine if different groups within the sample (e.g. emergency vs elective PCI, men vs women) with the same characteristics responds to a given item differently	Differential item functioning	Uniform DIF is indicated by a significant main effect for the person factor (e.g. sex) using a Bonferroni adjusted *p* value for significance [22]. Non-uniform DIF is indicated by a significant interaction effect [22].
Targeting	To determine the degree to which the PROM was targeted appropriately to patients following PCI (floor and ceiling effects)	Mean location score Person-item threshold distribution map	A mean logit score of zero indicates a well-targeted scale [22]. Items should be well-aligned with the full range of individual person scores for a well-targeted scale.

PROM, patient reported outcome measure; PCA, principal component analysis; DIF, differential item functioning; PSI, Person separation index

The scores from the final items that can be included in the MC-PROM were also compared with EQ-5D utility scores to determine convergent validity by using Pearson’s product-moment correlation coefficient or Spearman’s rank order correlation. All data were analysed using SPSS v24.0 (IBM Corporation, Armonk, New York). Rasch analysis was conducted using the RUMM2030 package with a partial credit model to allow thresholds to vary for each individual item (RUMM Laboratory Pty Ltd, Perth, Australia).

#### Sample size

In order to obtain an appropriate degree of precision from the Rasch analysis, we aimed to recruit a sample between 108 to 243 participants depending on whether the eight items from the DCE are targeted appropriately to our sample [31]. This will ensure that participant responses are appropriately distributed across the five response options [31].

## Results

### Participant characteristics

The telephone survey was administered to 200 consecutive patients that had a PCI at one of the four participating hospitals during their routine 30-day follow-up phone call with VCOR. The majority of participants were men (69%) with a mean age of 65 years, which is reflective of all patients included in VCOR [3]. Most presented with acute coronary syndrome (ACS) (68%), of which 37% had a ST-elevation myocardial infarction (STEMI) while 31% had non-ST elevation ACS. The characteristics of participants are presented in Table 2.

**Table 2:** Characteristics of participants following PCI procedure.

	All participants (*n* = 200)	
**Male, *n* (%)**	139	69
**Age, mean (SD)**	65.0	11.4
**Age group, *n* (%)**		
≤65 years	104	52
> 66 years	96	48
**Procedure type, *n* (%)**		
Urgent	135	68
Elective	64	32
**Clinical presentation, *n* (%)**		
STEMI	73	37
NSTEACS	62	31
Non-ACS	65	33
**Pre-procedural risk factors, *n* (%)**		
Diabetes	36	18
Peripheral vascular disease	4	2
Cerebrovascular disease	7	4
Previous CABG	12	6
Previous PCI	41	21
**Discharge medications, *n* (%)**		
Aspirin	189	95
Thienopyridine	76	38
Ticagrelor	116	58
**HRQOL 30 days post-procedure, mean (SD)**		
EQ-5D utility score	0.91	0.15
EQ-5D VAS	78.1	16.7

SD, standard deviation; STEMI, ST-elevation myocardial infarction; NSTEACS, Non-ST elevation acute coronary syndrome; ACS, acute coronary syndrome; CABG, Coronary Artery Bypass Graft; PCI, percutaneous coronary intervention; HRQOL, health-related quality of life; EQ-5D, Euroqol; VAS, visual analogue scale

### Acceptability and feasibility

Overall, data quality was good with no missing responses for the eight items. The majority of participants (*n* = 195; 98%) did not have any issues with the wording of the items (*“very clear and easy”*, *“understandable”*, *“short and blunt”*) or the 5-point rating scale. However, two participants reported that they found it hard to remember all the response options over the telephone (*“there were too many options”*, *“hard to remember the options”*). From the perspective of the VCOR data managers, most reported that participants answered the items easily (*n* = 195; 98%). They also felt that the time taken to complete the eight items as part of routine VCOR follow-up was acceptable (*n* = 198; 99%).

### Initial assessment of fit

Rasch analysis of the data for the eight items identified by the DCE showed a lack of fit to the Rasch model with a significant χ^2^ Item-Trait Interaction statistic (Table 3). Whilst no serious item (fit residual mean -0.20; SD 1.33) or person (fit residual mean -0.24; SD 1.01) misfit was observed, analysis of individual person statistics revealed that one person had a positive fit residual value above 2.5. Inspection of person-by-item responses indicated that an unexpected response was observed for item 3 (*I have felt concerned or worried about my heart problems*). Participants appeared to have difficulty responding to this item because two questions (i.e. concerned and worried) were embedded in this item. Local dependency was also observed between items 5 (*I have lacked the confidence to do my usual activities*) and 6 (*I have been physically unable to do my usual activities*). The response to item 5 appeared to be dependent on the response to item 6 (and vice versa) as indicated by a person-item residual correlation of 0.28 which is greater than the expected value of 0.20. Despite this, we found evidence to support unidimensionality of the eight-items, demonstrated by <5% significant *t*-tests (Table 3). All eight items seemed to measure the same underlying construct of recovery post-PCI.

**Table 3:** Overall Rasch model fit statistics and reliability of an eight item and five-item cardiac PROM^a^.

	Ideal	8-item PROM	5-item PROM
*Total item-trait interaction*			
Total item χ^2^		43.75	16.99
d*f*		16	10
*p*-value	>0.05	0.00	0.07
*Items*			
Fit residual (mean)	0	-0.20	0.38
Fit residual (SD)	<1.5	1.33	0.81
*Persons*			
Fit residual (mean)	0	-0.24	-0.24
Fit residual (SD)	<1.5	1.00	1.11
*Unidimensionality*			
Equating *t*-tests	<0.05	0.01	0.00
Binomial dimensionality test (CI)	(lower limit <0.05)	-	-
Person-item residual correlation	<0.2	>0.2 items 5 & 6	<0.2 all items
*Person separation index*^b^	>0.7	0.62	0.43
Equivalent Cronbach’s *α*	>0.7	0.77	0.65

^a^As analysed using RUMM2030 (Rumm Laboratory Pty Ltd., Perth) for Windows

^b^Rasch based reliability statistic (analogous to Cronbach’s *α*)

PROM, patient reported outcome measure; d*f*, degrees of freedom; CI, confidence intervals

The pattern of thresholds was examined to determine whether disordering may have affected overall model fit. We were unable to obtain threshold maps for all items due to disordered thresholds. Inspection of category probability curves for all items indicated that participants were not using the 5-point rating scale (never to always) in a consistent manner (S4 Fig). In particular, participants appeared to have difficulty distinguishing between the ‘never’ and ‘rarely’ response options, as well as the ‘often’ and ‘always’ response options.

### Modifications following initial item analysis

Based on the initial assessment of fit, the response format of the eight items was modified to a 3-point rating scale—never, sometimes and always. A 3-point scale was selected because participants in the focus groups and the clinical expert panel during Stage 2 of the mixed-methods project confirmed that the assignment of three levels to each item was appropriate [20]. As item 6 (*I have been physically unable to do my usual activities*) correlated with item 5 (*I have lacked the confidence to do my usual activities*), we deferred again to results from the focus groups and interviews conducted in Stage 2 to decide which item should remain. Participants in the focus groups reported that having the confidence to perform activities of daily living was an important aspect of their recovery post-procedure [20]. Thus, it was recommended that we retained only item 5 for the final analysis.

Two additional items were removed—item 3 (*I have felt concerned or worried about my heart problems*) and item 4 (*I have felt dizzy or light-headed*)—due to the double-barrelled nature of the questions. Both items also continued to display disordered thresholds despite modification of the response options. A slight disordering of thresholds was observed with item 2 (*I have felt shortness of breath when exerting myself*) when a 3-point rating scale was adopted. However, shortness of breath was reported to be an important indicator of recovery following PCI based on previous discussions with cardiologists, nurses and allied health professionals. It was therefore retained and the remaining five items (Fig 1) were subjected to Rasch analysis.

### Fit to the Rasch model following modifications

As shown in Table 3, the final five items met the Rasch model expectations (χ^2^ Item-Trait Interaction statistic *p* = 0.07), with no misfitting items or persons. All five items demonstrated unidimensionality with no local dependency, confirming the appropriateness of summing the items to obtain an overall score. Adequate response thresholds were also observed for all five items, following rescoring of the response options to a 3-point rating scale (S5 Fig). The person separate index (PSI) statistic was 0.43, with an equivalent Cronbach α of 0.65. Whilst this indicates low internal consistency reliability and that the items may not be assessing a single construct, we consider it to be acceptable given there are only five items [32]. It is also plausible that these five items may influence a person’s recovery following a PCI-procedure (i.e. causal indicators) and may therefore not correlate strongly with each other [33, 34].

The possibility of differences in responses to each item was explored for sex (male or female), age of participants (≤65 years or >66 years), and procedure type (elective or emergency PCI) by analysis of DIF using a Bonferonni adjusted *p* value of 0.005 (0.05/10). Measurement invariance was not evident for all five items, demonstrating that people with the same level of recovery post-PCI responded in a consistent manner to the items regardless of their sex, age group and whether they had an emergency or elective procedure. Inspection of the relationship between the distributions of persons relative to items, however, indicated suboptimal targeting of the five items (mean logit score -1.24). Ideally there should be an even spread of items matching participants’ level of recovery post-PCI, but there were no items assessing individuals at the higher end of the recovery spectrum (S6 Fig).

### Convergent validity

Summary statistics for each item included in the MC-PROM are presented in Table 4. The scores from each item were also summed to obtain an overall score, where higher scores indicate greater patient recovery. To establish convergent validity, the overall score from the final five items were correlated with the EQ-5D utility score. A moderately strong correlation with the EQ-5D utility score (Spearman’s *rho* 0.53; *p*<0.001) was observed, which was consistent with our expectations. It is worth noting that in this sample of participants there were a total of 67 (33%) missing responses across all five dimensions of the EQ-5D. There were also fewer participants who reported being unable to perform their usual activities (*n* = 1; 1%) and being extremely anxious or depressed (*n* = 2; 1%). Additionally, there were no participants who reported that they were confined to bed, were unable to wash or dress and had extreme pain or discomfort. In contrast, one participant reported they always felt pain or discomfort (1%), six always lacked confidence to perform their usual activities (3%) and eight were always unhappy (4%) on the MC-PROM.

**Table 4:** Summary statistics for items included in the Monash University cardiac PROM (MC-PROM).

Item	All participants (*n* = 200)		
	Mean (SD)	Total number of ‘Never’ responses, *n*(%)	Total number of ‘Always’ responses, *n*(%)
1. I have felt pain or discomfort when exerting myself	1.82 (0.40)	165 (83%)	1 (1%)
2. I have felt shortness of breath when exerting myself	1.60 (0.55)	126 (63%)	6 (3%)
3. I have lacked confidence to do my usual activities	1.71 (0.52)	147 (74%)	6 (3%)
4. I have felt unhappy	1.67 (0.55)	142 (71%)	8 (4%)
5. I have had trouble falling asleep or staying asleep	1.56 (0.62)	124 (62%)	13 (7%)
Overall PROM score*	8.35(1.72)		

PROM, patient-reported outcome measure; SD, standard deviation

*Overall score obtained by summing scores from each item with higher scores indicating better recovery

## Discussion

Although PCI is one of the most common medical procedures worldwide [4], little is known about how patients perceive their recovery following the procedure [5]. Existing PROMs that can be used within this population are either very long such as the CROQ-PTCA with 47 items, or may not be sufficiently sensitive to assess recovery post procedure, such as the EQ-5D [28]. Given the need for sound measurement of health-related outcomes from the patient’s perspective for this common procedure [14], we identified a succinct set of items that can be used as a PROM post-PCI that were derived using genuine patient input that included focus groups, interviews and a DCE. The MC-PROM (Fig 1) demonstrated adequate measurement properties and is brief, which enhances its utility within the context of a clinical registry and for clinical practice purposes. Importantly, the MC-PROM was also robust across elective and emergency cohorts of patients.

Rasch analysis was used to refine the symptoms and feelings that could be included in a PROM to assess recovery following a PCI. The Rasch measurement model is recognised as a preferred method for psychometric evaluations of outcome measures, particularly when developing a new measurement instrument [21, 23]. This is because the model allows measurement issues such as response thresholds or measurement invariance to be easily identified in comparison to traditional methods [23, 33]. By using Rasch analysis and consulting with clinical experts, we were able to validate and refine the items for a PROM by modifying the response options and selecting items that fit the model expectations [23, 35]. As a result, we found good structural validity for the final MC-PROM. All five items demonstrated unidimensionality with no local dependency, and it therefore is appropriate to sum the items to obtain an overall score of health and wellbeing [23]. The MC-PROM also provides unbiased estimates of health and wellbeing across sex, age and procedure type, which means that valid comparisons can be made across different sub-groups of individuals following a PCI [33]. One of the strengths of this study was the inclusion of acceptability and feasibility information. Given that data managers and participants found it easy to complete the MC-PROM, we believe it can be administered effectively within a clinical registry such as VCOR or other clinical setting with minimal respondent burden.

We did observe suboptimal targeting of the MC-PROM in the Rasch analysis, where there was an apparent ceiling effect. The final five items may not adequately capture the recovery of individuals who were at the better end of the health spectrum. This may have implications on whether it can detect clinically important changes in recovery following a PCI when people have lower scores (i.e. better recovery) [33]. The ceiling effect observed may have been influenced by the small number of items (*n* = 5) included in the MC-PROM. While the risk of floor and ceiling effects may be minimised if a greater number of items were included, this would reduce the acceptability and utility of the MC-PROM to be integrated into a clinical registry. Given the need to use valid and reliable instruments that have minimal burden on patients and healthcare teams [6], it may be worth rewording existing items to improve the measurement of recovery for those who have lower scores in a further validation study. Nevertheless, the five items included in the MC-PROM appears to have a broader scale width compared to the EQ-5D—70% of participants reported having no issues compared to 78% of who reported having no problems on the EQ-5D. It also had fewer missing responses. This suggests that for a clinical registry such as VCOR, the MC-PROM may provide a better indication of patients’ perceptions about their recovery.

It is important to acknowledge that the sample for this analysis consisted predominantly of patients who had a PCI due to ACS. There is therefore limited generalisability to individuals who have a PCI for elective non-ACS indications. Nevertheless, it is encouraging to note that all five items did not demonstrate measurement invariance for the type of procedure (i.e. elective or urgent PCI) in the Rasch analysis. This suggests that irrespective of whether patients had an elective or urgent PCI, they responded to the PROM items consistently in the same way [22].

The key attributes of a high-quality PROM are that qualitative methods are applied to ensure that the domains and item wording reflect the patient’s preferences, and that item response theory techniques are used to ensure that meaningful inferences can be made [14, 36]. It is also imperative that the PROM is brief and simple to administer, complete and score to minimise the burden on patients, clinicians and researchers [14]. The final set of items identified in this study have been developed specifically for patients following PCI, with items derived from focus groups and interviews with patients [20, 28]. In addition, this is the first Rasch-tested PROM for this population. As it only contains five items, the MC-PROM can be easily administered via the phone, thereby meeting the requirements for a high-quality PROM that can be used in a clinical cardiac registry or in the clinical care setting.

Further work is needed to determine whether the MC-PROM can be used together with other clinical indicators to predict the quality of care following a PCI and other cardiac interventions [37]. This includes refining or rewording items such as item 3 to determine whether it may improve the internal consistency and overall targeting of the PROM. We also recommend further psychometric evaluations, in particular formal testing of its criterion validity (e.g. comparing PROM scores between those with and without ischemic heart disease), reproducibility (e.g. test-retest survey) and responsiveness in a larger and more heterogenous sample. Finally, it may be beneficial to examine the validity of a written version of the PROM so that it can be self-completed by patients in a clinical setting.

## Conclusion

This study has identified five items that best form a concise and psychometrically sound PROM to assess the recovery of patients post-PCI. The MC-PROM has the potential to improve the quality of patient care and identify opportunities to improve care models and patient-centred care, such as enabling the systematic identification of patients who may need further assessment for depression or for cardiac rehabilitation. A patient-derived measure of post-PCI recovery is an important complement to current clinical outcome information, which currently focuses mainly on rare adverse clinical events, and therefore provides a more holistic approach to managing the cardiac patient post-procedure.

## Supporting information

S1 Fig A mixed methods approach to develop and refine a new cardiac patient-reported outcome measure for people following percutaneous coronary interventions. (DOCX)

S2 Fig Example of a choice set participants were asked to select in the discrete choice experiment [28]. (DOCX)

S3 Fig The eight outcomes identified from the discrete choice experiment that were most valued by patients following percutaneous coronary interventions. (DOCX)

S4 Fig Category probability curves for the eight items included in the cardiac patient-reported outcome measure. (DOCX)

S5 Fig Response thresholds for the final five items included in the cardiac patient-reported outcome measure. (DOCX)

S6 Fig Person-item threshold distribution depicting targeting for five items included in the cardiac patient-reported outcome measure. Distributions of the locations of people and items on the common logit metric (negative values = good health and wellbeing; positive values = poor health and wellbeing) are depicted on the upper and lower panels respectively.
(DOCX)

S1 Table Symptoms and feelings identified to be important outcomes post percutaneous coronary interventions. (DOCX)
