# Supplementary material for: What matters most to patients following percutaneous coronary interventions? A new patient-reported outcome measure developed using Rasch analysis
Source: PLoS One. 2019 Sep 5;14(9):e0222185. doi: 10.1371/journal.pone.0222185 (PMC6728040; doi:10.1371/journal.pone.0222185)
Supplement: S1 Fig — (DOCX) [file pone.0222185.s001.docx]

**S1 Fig. A mixed methods approach to develop and refine a new cardiac patient-reported outcome measure for people following percutaneous coronary interventions.**
