# Supplementary material for: What matters most to patients following percutaneous coronary interventions? A new patient-reported outcome measure developed using Rasch analysis
Source: PLoS One. 2019 Sep 5;14(9):e0222185. doi: 10.1371/journal.pone.0222185 (PMC6728040; doi:10.1371/journal.pone.0222185)
Supplement: S2 Fig — (DOCX) [file pone.0222185.s002.docx]

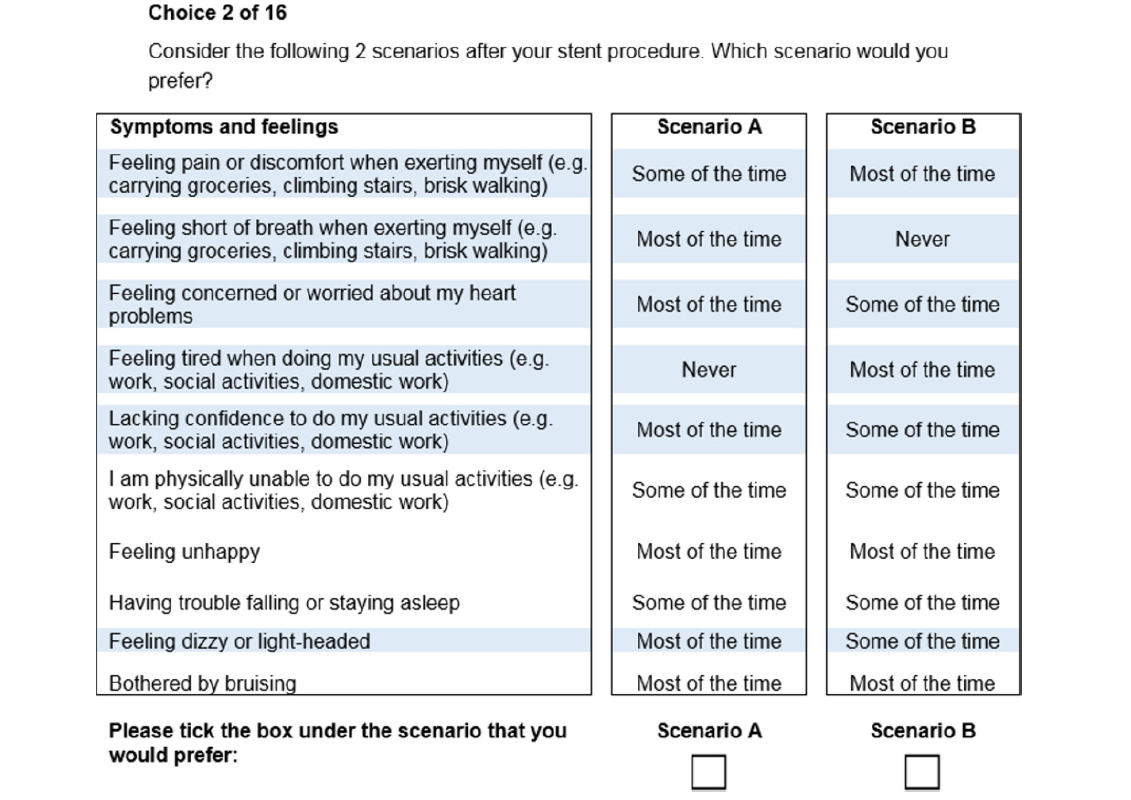


**S2 Fig. Example of a choice set participants were asked to select in the discrete choice experiment [24].**
