# Supplementary material for: What matters most to patients following percutaneous coronary interventions? A new patient-reported outcome measure developed using Rasch analysis
Source: PLoS One. 2019 Sep 5;14(9):e0222185. doi: 10.1371/journal.pone.0222185 (PMC6728040; doi:10.1371/journal.pone.0222185)
Supplement: S5 Fig — (DOCX) [file pone.0222185.s005.docx]

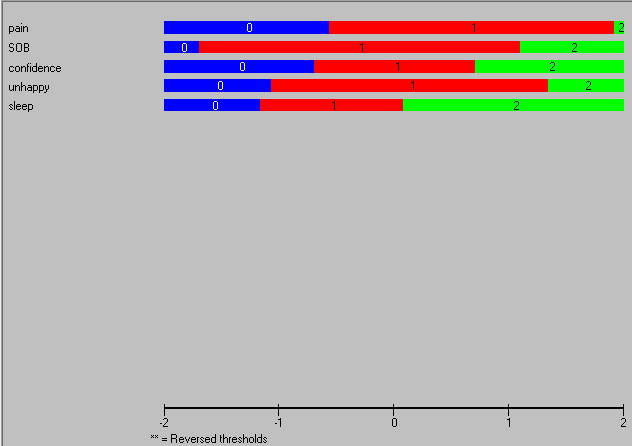


**S5 Fig. Response thresholds for the final five items included in the cardiac patient-reported outcome measure.**
