# Supplementary material for: What matters most to patients following percutaneous coronary interventions? A new patient-reported outcome measure developed using Rasch analysis
Source: PLoS One. 2019 Sep 5;14(9):e0222185. doi: 10.1371/journal.pone.0222185 (PMC6728040; doi:10.1371/journal.pone.0222185)
Supplement: S6 Fig — Distributions of the locations of people and items on the common logit metric (negative values = good health and wellbeing; positive values = poor health and wellbeing) are depicted on the upper and lower panels respectively. (DOCX) [file pone.0222185.s006.docx]

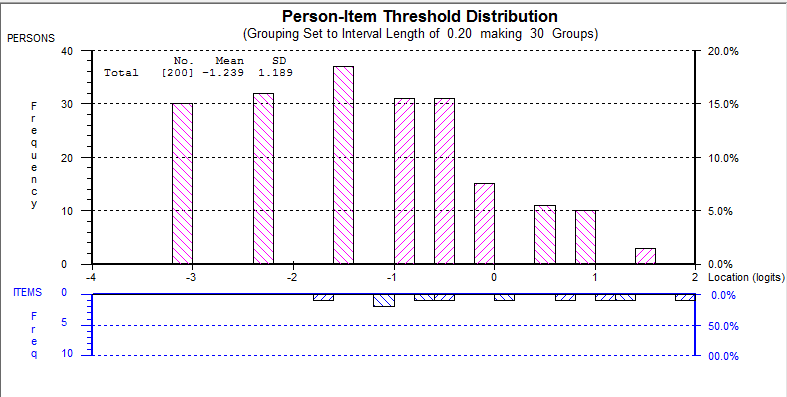


**S6 Fig. Person-item threshold distribution depicting targeting for five items included in the cardiac patient-reported outcome measure.** Distributions of the locations of people and items on the common logit metric (negative values = good health and wellbeing; positive values = poor health and wellbeing) are depicted on the upper and lower panels respectively.
