# Supplementary material for: What matters most to patients following percutaneous coronary interventions? A new patient-reported outcome measure developed using Rasch analysis
Source: PLoS One. 2019 Sep 5;14(9):e0222185. doi: 10.1371/journal.pone.0222185 (PMC6728040; doi:10.1371/journal.pone.0222185)
Supplement: S1 Table — (DOCX) [file pone.0222185.s007.docx]

**S1 Table. Symptoms and feelings identified to be important outcomes post percutaneous coronary interventions.**

| **Item** | **Level 1 (worse)** | **Level 2** | **Level 3 (better)** |
| --- | --- | --- | --- |
| Pain or discomfort on exertion (e.g. carrying groceries, climbing stairs, brisk walking) | Most of the time | Some of the time | Never |
| Shortness of break on exertion (e.g. carrying groceries, climbing stairs, brisk walking) | Most of the time | Some of the time | Never |
| Concerned or worried about heart problem | Most of the time | Some of the time | Never |
| Tiredness when doing usual activities (e.g. work, social activities, domestic work) | Most of the time | Some of the time | Never |
| Physically unable to do usual activities (e.g. work, social activities, domestic work) | Most of the time | Some of the time | Never |
| Feeling unhappy | Most of the time | Some of the time | Never |
| Trouble falling or staying asleep | Most of the time | Some of the time | Never |
| Dizziness or light-headedness | Most of the time | Some of the time | Never |
| Bruising | Most of the time | Some of the time | Never |
